# An Interesting Case of Poisoning by Tyrotoxicon

**Published:** 1887-12

**Authors:** 


### *AN INTERESTING CASE OF POISONING BY TYROTOXICON.*

Dr. V. C. Vaughan made a verbal report to the Michigan State Board of Health, relative to tyrotoxicon poisoning in Milan, Mich., which presents some new features. He was called, September 25th, to see the family, two members of which were dying, and two others were seriously ill, while one other, a daughter twenty years old, who had been away for some months, was not sick. The father had been sick about eight days, and the three others for a shorter time. The attending physician, suspecting that the sickness was due to tyrotoxicon, had warned the daughter, when she returned, not to drink milk. She complied, and was not sick. The mother and son died. He found the four patients breathing in a labored manner, the pupils of the eyes were dilated, and throbbing of the abdominal aorta was marked. The son was more or less delirious. All would pass urine involuntarily, but constipation was marked. Unfortunately, there was no thorough examination of the body after the first death; but, upon the second death, a thorough *post mortem* examination was held. Nothing abnormal was found, except that the large intestine was constricted by its circular fibres, which accounted for the constipation; the heart was arrested in systole; the death occurred from failure of the heart. The analysis of the stomach, by Prof. Prescott, revealed no mineral poison. Dr. Vaughan's assistant, Mr. Novi, found enough tyrotoxicon in the contents of the stomach and intestines to sicken a cat.

Dr. Vaughan had confirmed the diagnosis of the attending physician, who said it was sickness due to "cheese poison." It was not like arsenic poisoning, and, although it somewhat resembled poisoning by belladonna, yet there were points of dissimilarity enough to throw that out as a possible cause, especially as another poison was actually found in the stomach.

Dr. Vaughan soon made up his mind that the sickness was probably due to the bad and unwholesome condition of the house, which was fifty years old and nearly rotten. One floor was nearly rotted away, and was covered by a newer one. The house had settled a good deal; there was no cellar; the land in all directions sloped towards the house, so that the building was constantly on damp soil, as there was no artificial drainage. The sweepings and moppings for years had accumulated in the cracks of the floor, so that, when the floor was taken up, a nauseating odor arose. The farmer sold cream to a creamery in the neighborhood, the proprietor of which had received the documents of the Michigan State Board of Health on cholera infantum and poisoning by cheese, milk, etc. He induced the farmer to keep his milk away from the house, and in a cool place, until the cream was collected and taken away. The milk consumed by the four members of the family was kept in a small closet or pantry in the house, where they frequently went and helped themselves to milk. They had been sick in the same way a number of times before this violent outbreak, which resulted in the death of two of their number. Dr. Vaughan made experiments as follows: He placed fresh milk in the pantry for a short time, and then found enough tyrotoxicon had developed in the milk to make a cat sick. He took some of the earth under the pantry floor, and placed a small quantity of it in some fresh milk, soon after which tyrotoxicon was obtained from the milk, while none could be obtained from another sample of fresh milk which stood by the side of the milk in which the earth had been placed. This seems to demonstrate that the soil contained the germ of decomposition which produces the poison.—*Medical Record.*

